# Healthcare Utilization and Inequalities in Breast Cancer Early Detection Behaviors Among Women in Türkiye: A Nationally Representative Cross-Sectional Study

**DOI:** 10.3390/healthcare14162516

**Published:** 2026-08-12

**Authors:** Bestegül Çoruh Akyol, Latife Merve Yildiz, Yasemin Kiliç Öztürk, Özgür Enginyurt

**Affiliations:** 1 Family Medicine Department, Faculty of Medicine, Ordu University, Ordu 52200, Türkiye; latifeyaglioglu05@gmail.com (L.M.Y.); enginyurt72@gmail.com (Ö.E.); 2 Department of Family Medicine, Tepecik Training and Research Hospital, Izmir Faculty of Medicine, University of Health Sciences Türkiye, İzmir 35170, Türkiye; dryko38@gmail.com

**Keywords:** breast neoplasms, early detection of cancer, early diagnosis, mammography, awareness, preventive medicine

## Abstract

**Highlights:**

**What are the main findings?**

- Breast cancer early detection behaviors were closely associated with contact with the health system, in addition to individual characteristics.
- The combined use of modified Poisson regression and CART analyses identified not only independent correlates but also population subgroups requiring priority intervention.

**What are the implications of the main findings?**

- Strategies aimed at increasing screening participation should be planned using holistic approaches that focus on strengthening engagement with the healthcare system. Reminder systems, opportunistic recommendations during routine visits, and targeted invitation strategies may help translate healthcare contact into higher screening participation.
- The study contributes to the development of evidence-based screening strategies at the national level while offering actionable insights for health systems with similar delivery models.

**Abstract:**

Background/Objectives: The aim of this study is to determine the extent to which early breast cancer detection behaviors in Türkiye are associated with age, education, income, and chronic disease status, and to evaluate the relationship of healthcare service utilization (contact with family physicians/general practitioners and specialists) with these behaviors. Methods: A cross-sectional secondary data analysis was conducted using nationally representative microdata from the Turkish Statistical Institute’s (TUIK) 2022 Turkiye Health Survey. The analysis included 5554 women aged 40–69. Descriptive statistics were calculated using the FERTFACTOR sample weight. Fully adjusted modified Poisson regression models were constructed for recent mammography (within the last 2 years) and monthly breast self-examination (BSE) (adjusted prevalence ratio, aPR), while Classification and Regression Tree (CART) analysis with five-fold cross-validation was applied to identify targetable risk profiles. (The maximum tree depth was set at 3, and the minimum terminal node size at 250 individuals.) Results: The rates of undergoing a recent mammogram and performing monthly BSE were 23.5% and 25.0%, respectively. Both early detection behaviors were more frequent among women who had contact with both a family physician/general practitioner and a specialist physician within the past 12 months. While contact with a specialist physician was independently associated with both behaviors, education level was positively associated with them, whereas the presence of a chronic disease was associated only with having a recent mammogram. In the CART analysis, the initial split occurred based on BSE behavior, and the prevalence of recent mammography varied approximately eightfold across the terminal nodes (cross-validated AUC = 0.681). Conclusions: In Türkiye, behaviors related to the early detection of breast cancer are associated with age, education, and contact with healthcare services. Fewer than one in four women were up to date with mammography, underscoring a substantial early detection gap. Contact with a specialist physician showed the strongest association; however, given the cross-sectional design, causality cannot be inferred and reverse causation cannot be excluded. Systematizing early detection recommendations within primary care and implementing targeted strategies for low-socioeconomic groups may help increase participation in screening.

## 1. Introduction

Breast cancer is the most frequently diagnosed malignancy among women globally and stands as a major public health issue due to the disease burden it imposes [1]. According to GLOBOCAN estimates for 2022, approximately 2.3 million women worldwide were newly diagnosed with breast cancer, and around 670,000 women lost their lives to the disease [2]. Early diagnosis and the effective implementation of appropriate screening programs in breast cancer management are recognized as fundamental public health strategies for reducing disease-related mortality, increasing the likelihood of early-stage diagnosis, enabling the use of less aggressive and cost-effective treatment approaches, and improving quality of life [3,4]. Strategies for the early detection of breast cancer encompass fostering breast awareness, evaluating symptoms without delay, and implementing evidence-based mammography screening programs for the appropriate age group [5]. Participation rates in screening programs vary significantly across countries; according to the global CanScreen5 database, mammography coverage ranges from 1.7% (Bangladesh) to 85.5% (United Kingdom) [6]. In our country, within the scope of the national cancer screening program, biennial mammography screening is conducted for women aged 40–69, and periodic physician examinations are performed based on age group [7].

The determinants of breast cancer screening behaviors have been extensively studied in the literature. A recent, comprehensive systematic review indicates that advanced age, higher levels of education and income, marital status, and regular access to healthcare services consistently predict participation in screening [8]. Andersen’s model of health care utilization behavior presents these determinants within a conceptual framework that classifies them as predisposing factors (age, education, marital status), enabling factors (income, health insurance, access to services), and need factors (chronic illness, perceived general health) [9]. In this context, the utilization of healthcare services itself—particularly through a physician’s recommendation for screening—can function as a facilitating mechanism; systematic reviews indicate that physician recommendations consistently increase screening participation [10]. To the best of current knowledge, the majority of studies examining breast cancer screening behaviors have been single-center or regional in scope, or have focused on knowledge, attitudes, and awareness within specific populations. Studies evaluating socioeconomic characteristics, health status, and healthcare utilization together at the national level are limited. In particular, previous national analyses have generally treated healthcare utilization as a single dichotomous indicator, without differentiating contact with family physicians/general practitioners from contact with specialist physicians, and without examining how sociodemographic characteristics and healthcare contact combine to define subgroups of women with distinct screening profiles. Conventional regression models estimate the independent association of each factor but do not readily identify such subgroups; tree-based partitioning methods complement regression by hierarchically dividing the population into mutually exclusive, directly targetable risk profiles. This study aims to evaluate—at the national level—the sociodemographic, health-related, and healthcare utilization determinants of early breast cancer detection behaviors among women in Türkiye using fully adjusted multivariate models, and to identify subgroups of women with similar risk profiles through Classification and Regression Tree (CART) analysis.

## 2. Materials and Methods

### 2.1. Dataset and Ethics Committee

The data employed in this research were of a secondary nature, drawn from the Türkiye Health Survey Research micro datasets provided by the Turkish Statistical Institute (TÜİK) [11]. These datasets are made available to researchers upon request and the payment of a specified fee. Since the data are anonymized, no personally identifiable details are revealed, thereby upholding participant confidentiality. As a result, this study utilized a pre-existing, publicly accessible secondary dataset, and did not necessitate any further consent or ethical approval. The study was conducted in accordance with the Declaration of Helsinki.

### 2.2. Study Sample and Design

Two modules were derived from the 2022 Türkiye Health Survey (THS) microdata set. The resulting models consist of (i) a general module comprising women aged 18 and older (n = 11,161) and (ii) a subsample (n = 5554) corresponding to the 40–69 age range—the target population for mammography screening under Türkiye’s National Cancer Control Program—and including questions on breast cancer screening behavior. The analytical sample was restricted to this 40–69 age subsample, and descriptive analyses were conducted on the 5554 women. One participant with missing data on the income variable was excluded from the fully adjusted multivariate regression models; consequently, these models were run on a sample of 5553 women.

The dependent variables were defined as binary (present/absent): (i) recent mammography (having had a mammogram within the past 2 years) and (ii) monthly breast self-examination (BSE) (reporting BSE frequency as “once a month”). The survey recorded whether and when mammography had been performed, but not the indication for the examination; consequently, screening mammography could not be distinguished from diagnostic mammography, and the mammography outcome reflects overall (screening or diagnostic) utilization. For the purposes of sensitivity analysis and descriptive purposes, alternative time windows—such as mammography within the past 12 months, mammography within the past 3 years, lifetime mammography, and any BSE—were also reported at the descriptive level.

The independent variables include age group (40–49/50–59/60–69), education level (no schooling/primary school/secondary education/high school/university and above), income group (low/medium/high), marital status, Social Security Institution (SGK) coverage, presence of chronic disease, general health perception, tobacco/alcohol/e-cigarette use, fruit and vegetable/salad consumption, sleep problems, and contact with health services in the last 12 months. Health service contact was addressed as a four-category variable (no contact/family physician only/specialist only/both) and also through a second parameterization in which contact with a family physician and contact with a specialist were included in the same model as two separate binary indicators. Because this was a secondary analysis of a general-purpose health survey, the analysis was necessarily restricted to the variables available in the dataset; behavioral and psychosocial factors such as physician recommendation of screening, health literacy, perceived breast cancer risk, family history of breast cancer, and receipt of a screening invitation were not collected in the survey and could not be examined.

### 2.3. Statistical Analysis

In descriptive analyses, percentages were calculated using the FERTFACTOR sample weight provided by TUIK, while the number of individuals in the tables is presented as unweighted n. It is known that odds ratios derived from logistic regression tend to overestimate prevalence ratios for common binary outcomes in cross-sectional data; therefore, fully adjusted modified Poisson regression models (Poisson regression with robust variance estimation) were constructed to directly estimate adjusted prevalence ratios (aPR) for up-to-date mammography and monthly BSE [12]. The models were adjusted for age, education, income, marital status, social security coverage (SGK), chronic disease, general health status, tobacco use, alcohol use, e-cigarette experience, fruit consumption, vegetable/salad consumption, and sleep problems; weights were normalized to the sample size based on FERTFACTOR. Multicollinearity among the explanatory variables was assessed by calculating variance inflation factors (VIF) for the full covariate set; all VIF values were below 2.5 (maximum, 2.4; mean, 1.6), indicating no substantial collinearity. Influential observations were examined using Cook’s distance; the maximum value was below 0.01, indicating that no individual observation exerted undue influence on the estimates. Overall model adequacy was further evaluated by re-estimating the fully adjusted models under an alternative link specification (log-binomial regression, described below) and verifying the stability of the estimates across specifications.

To identify the hierarchical relationship between variables and the subgroups with the highest and lowest screening probabilities, a CART decision tree analysis was conducted with “recent mammography” as the target variable; this approach complements traditional regression and is increasingly used in the literature to hierarchically model the determinants of screening behaviors using data from nationally representative health surveys [13]. CART was preferred over more complex machine learning algorithms (e.g., random forests, gradient boosting, or neural networks) because it yields a single, transparent set of hierarchical decision rules that can be inspected directly and translated into practice; such interpretability is particularly valuable for public health decision making, where clearly defined target groups are more actionable than marginal gains in predictive accuracy. The main limitations of CART—instability to small perturbations of the data and typically moderate discriminatory performance—were addressed by pre-specifying conservative complexity constraints (maximum tree depth of 3; minimum terminal node size of 250) and by evaluating performance with five-fold cross-validation. Variables included in the model were BSE, contact with a specialist/physician, contact with a family physician/general practitioner, age, education, income, marital status, social security coverage, chronic disease, general health status, and lifestyle factors; the Gini index was used as the splitting criterion, with a maximum tree depth of 3 and a minimum terminal node size of 250 individuals. The model’s discriminatory power was evaluated using the apparent AUC and the AUC from 5-fold cross-validation.

To assess the robustness of the models, the fully adjusted Poisson models were re-run using log-binomial regression, and the direction, magnitude, and significance of the resulting prevalence ratios were compared; no substantial difference was observed between the two methods. Given the simultaneous testing of multiple variables, the risk of Type I error was taken into account when interpreting marginally significant findings (0.01 < *p* < 0.05), and such findings were evaluated with caution; the main conclusions were based on associations where confidence intervals were clearly distant from 1 and significance was at the *p* < 0.01 level.

In all analyses, *p* < 0.05 was considered statistically significant. Analyses were conducted using Python 3.10.12 (pandas, statsmodels, and scikit-learn libraries). Since the findings were derived from cross-sectional data, relational terms such as “was associated with” or “accompanied by” were preferred over causal statements in the text.

## 3. Results

### 3.1. Analytical Sample and Early Detection Behaviors

A total of 5554 women aged 40–69 were included in the analysis. The rates of undergoing a recent mammogram, performing monthly BSE, and performing BSE at any time were calculated as 23.5%, 25.0%, and 57.8%, respectively (Table 1).

Regarding mammography, 45.6% of the participants reported never having undergone the procedure. As for breast self-examination (BSE), the proportion of those who never performed it was 42.2%, while the rate of monthly BSE was found to be 25.0% (Table 2).

### 3.2. Healthcare Service Contact and Early Detection Behaviors

**Figure 1:**
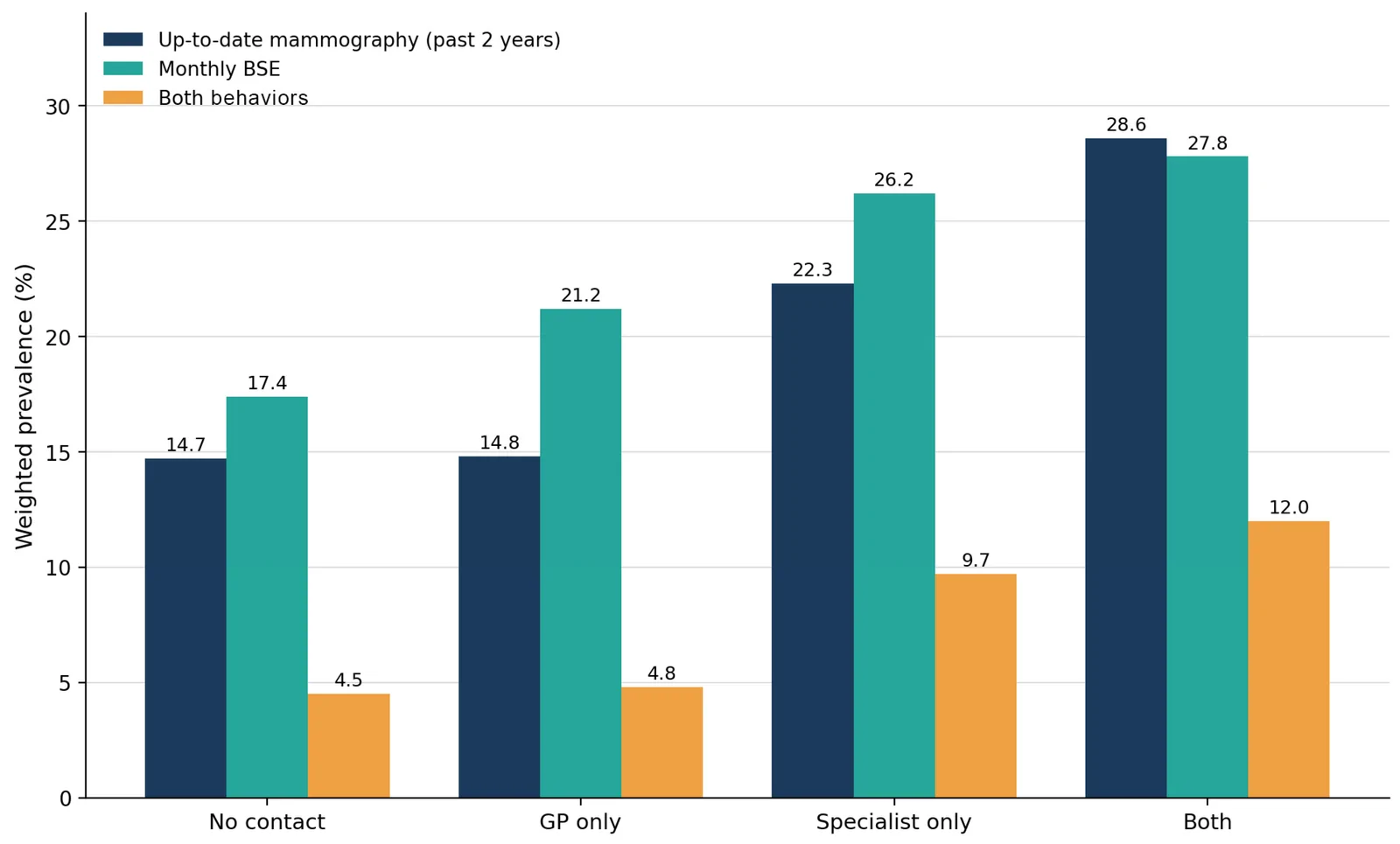
Prevalence of up-to-date mammography, monthly BSE and the combined behavior according to healthcare contact (Values are weighted percentages. Up-to-date mammography was defined as having undergone mammography within the previous 2 years, and monthly BSE as reporting a BSE frequency of once a month).

Both early detection behaviors increased in a graded manner across healthcare contact categories, from 14.7% (no contact) to 28.6% (contact with both) for up-to-date mammography and from 17.4% to 27.8% for monthly BSE (Figure 1).

### 3.3. Multivariable Models

In fully adjusted models, contact categories involving a specialist/medical department (specifically “specialist only” and “both”) were positively associated with both recent mammography and monthly BSE, whereas contact with a family physician/general practitioner alone was not associated with mammography and showed only borderline significance regarding BSE. Education level demonstrated a graded positive association with both behaviors; high income was associated only with BSE, and chronic illness only with mammography. BSE rates were lower in the 60–69 age group (Table 3).

When contact with a family physician/general practitioner and contact with a specialist were included in the same model as two separate indicators, both types of contact remained positively associated with both behaviors, and the associations were consistently stronger for specialist contact (up-to-date mammography: aPR 1.66 vs. 1.18; monthly BSE: aPR 1.30 vs. 1.12) (Table 4).

### 3.4. Behavioral Phenotypes Regarding BSE and Mammography

**Figure 2:**
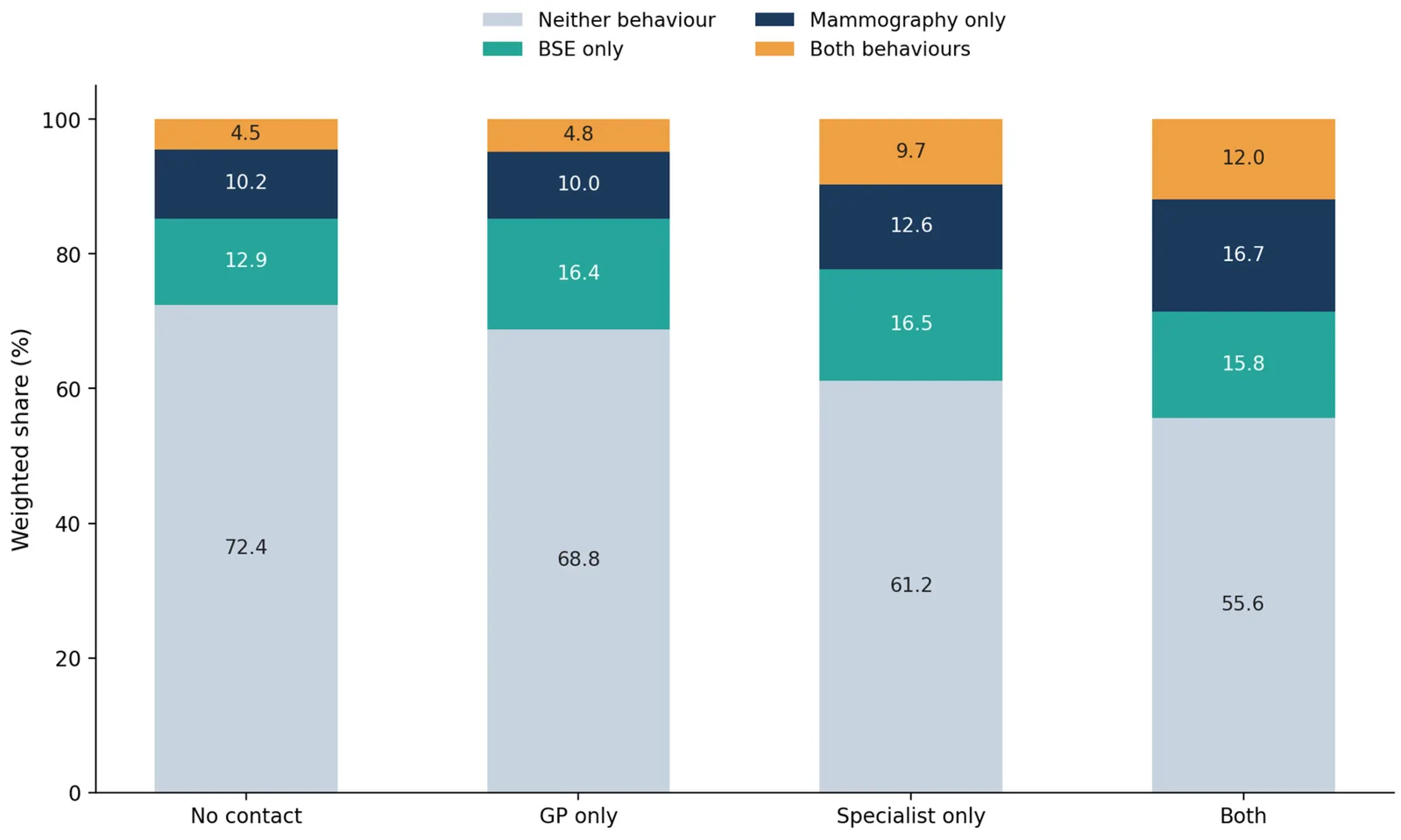
Phenotypes of monthly BSE and up-to-date mammography according to healthcare contact (Phenotypes were derived from monthly BSE status and mammography within the past 2 years. Values are weighted percentages).

When the distribution of phenotypes in the total sample was examined, the phenotype characterized by the absence of either behavior accounted for the largest share in the group with no history of contact; conversely, the phenotype involving both behaviors increased in prevalence alongside the level of contact, reaching its highest value in both contact groups (Figure 2).

### 3.5. CART Decision Tree Analysis

**Figure 3:**
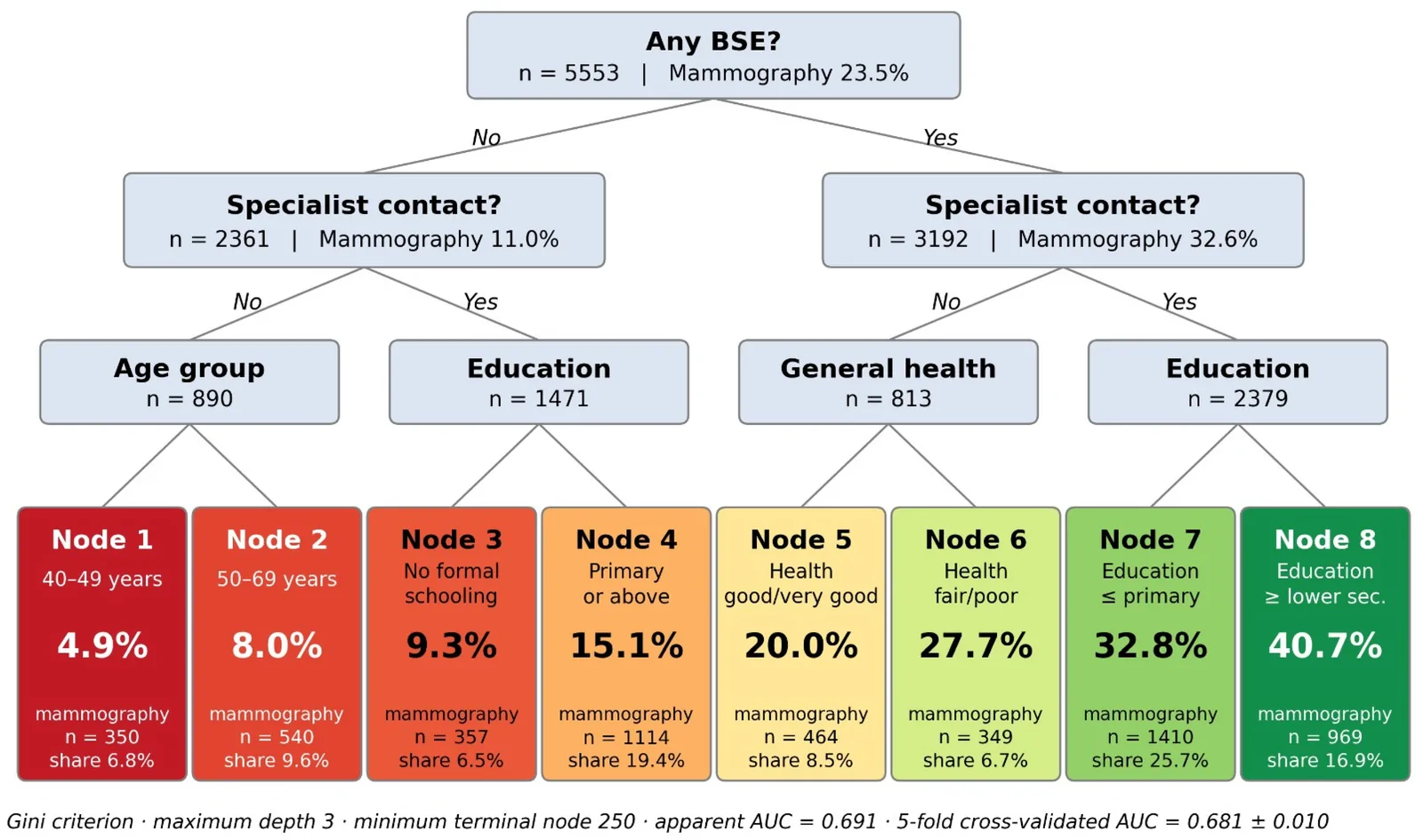
Classification and regression tree (CART) for up-to-date mammography (Terminal node boxes display the node number, the weighted prevalence of up-to-date mammography, the unweighted number of participants (n) and the weighted node share; box colour is graded from low (red) to high (green) prevalence. Values in the internal nodes indicate the n and the weighted mammography prevalence within that node. In this model, BSE was defined as any BSE, and specialist contact refers to a physician visit within the past 12 months. The model was grown using the Gini criterion (maximum depth, 3; minimum terminal node size, 250). Apparent AUC = 0.691; 5-fold cross-validated AUC = 0.681 ± 0.010).

In the full CART model, the initial split was based on the BSE variable; subsequent splits occurred according to specialist/physician contact and age for those who did not practice BSE, and according to specialist/physician contact, general health status, and education level for those who did. Current mammography prevalence in the terminal nodes varied approximately eightfold; the lowest prevalence was observed in the 40–49 age group who did not practice BSE and had no specialist/physician contact, while the highest prevalence was found among women who practiced BSE, had specialist/physician contact, and possessed a secondary education or higher (Figure 3).

**Figure 4:**
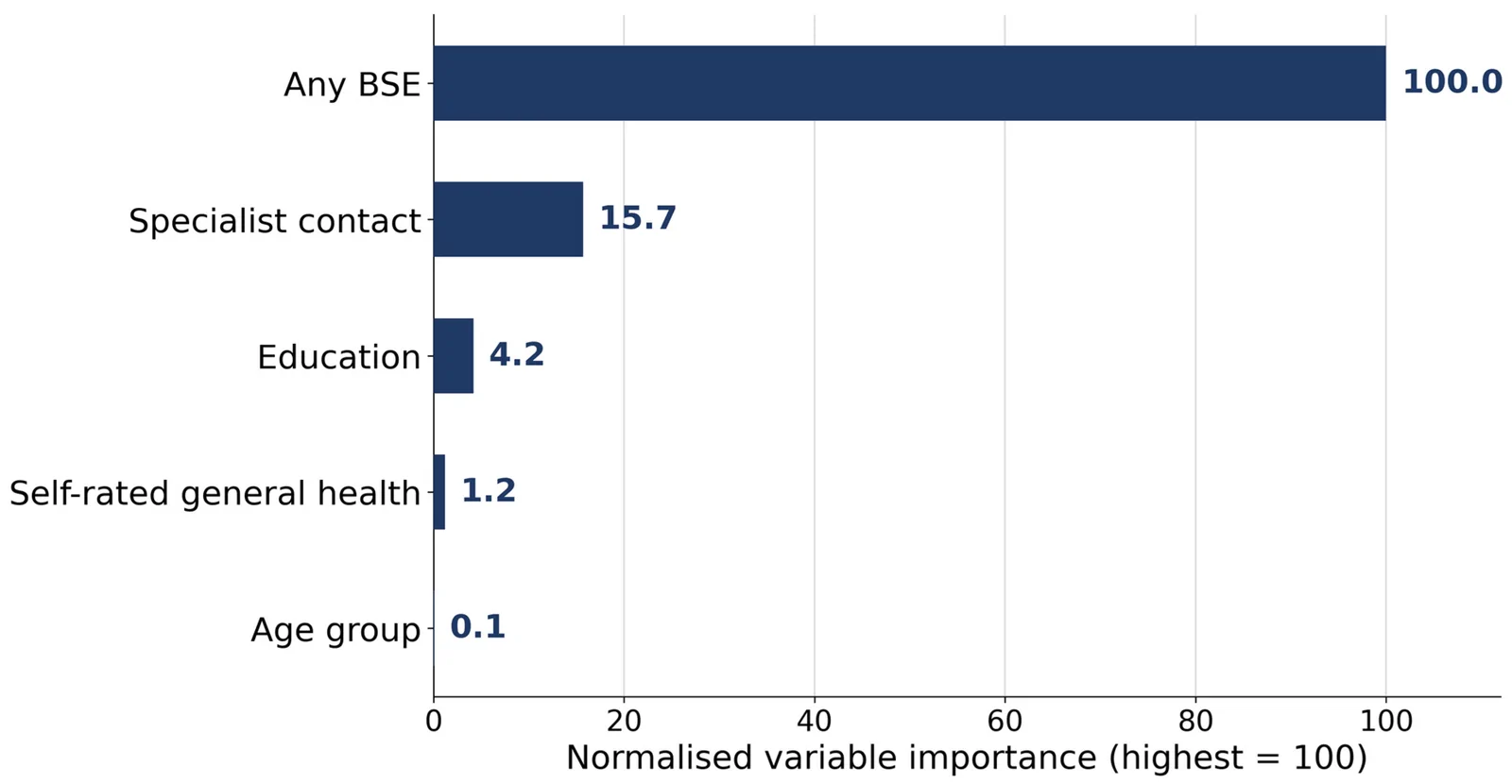
Normalised variable importance in the CART model (The highest importance score was set to 100. Only variables with non-zero importance are displayed. All remaining candidate variables (GP contact, income, marital status, SSI coverage, chronic disease, tobacco use, alcohol use, e-cigarette experimentation, fruit consumption, vegetable/salad consumption and sleep problems) had an importance of zero and were not selected for any split in the tree).

In the CART model, the highest normalized importance levels were calculated for the “any BSE” and “specialist/branch” variables; these were followed by education, general health, and age. The importance levels for the “family physician/general practitioner” variable, SGK status, and marital status were zero, and these variables did not appear in any splits within the tree (Figure 4).

## 4. Discussion

This study is one of the few to evaluate breast cancer early detection behaviors in Türkiye at a national level while simultaneously addressing both breast self-examination and current mammography practices. It assessed the relationship of sociodemographic characteristics and healthcare utilization with these behaviors using multivariate models; furthermore, through CART analysis, it identified subgroups of women with distinct risk profiles, thereby evaluating early detection behaviors not merely in terms of screening participation, but also regarding healthcare contact patterns and behavioral phenotypes. This approach enabled not only the identification of independent determinants but also the definition of risk profiles that could inform the planning of targeted preventive healthcare services.

This study found that approximately half (45.6%) of the women had never undergone a mammogram in their lifetime, while 42.2% had never performed BSE. Conversely, only one in four women was found to perform BSE regularly on a monthly basis. These findings align with previous national data indicating that early detection behaviors for breast cancer in Türkiye have not yet reached desired levels [14]. Similarly, studies conducted in various regions of Türkiye have reported that, despite women possessing relatively adequate knowledge and awareness regarding breast cancer, the frequency of regular BSE practice and participation in mammography screenings remains insufficient [15,16,17]. International systematic reviews also demonstrate that disparities in socioeconomic conditions and access to healthcare services significantly influence screening behaviors and hinder the achievement of recommended screening rates [18,19].

This study determined that education level is positively and incrementally (in a dose–response-like manner) associated with both regular BSE and up-to-date mammography screening; conversely, high income was found to be associated only with regular BSE, while the presence of a chronic disease was associated only with up-to-date mammography. Furthermore, an analysis of healthcare utilization variables revealed that contact with a specialist physician, in particular, was more strongly associated with both early detection behaviors than contact with a family physician or general practitioner. As the dataset did not include information regarding the specialist’s medical specialty or whether the consultation was breast-related, it is impossible to distinguish whether the observed association stemmed from a referral specifically targeting breast health or from general health-seeking behavior. Likewise, because the indication for mammography was not recorded, screening examinations cannot be separated from diagnostic examinations performed to investigate symptoms; some of the mammograms captured by the outcome will therefore have followed, rather than preceded, contact with a specialist, which would further inflate the observed association. On the other hand, part of the strong association with specialist contact may stem from reverse causality: women undergoing mammography or experiencing breast symptoms may have already been referred to or sought out a specialist; in this case, it may be the screening process—rather than contact—that led to the visit. Furthermore, women who consult a specialist may represent a group that uses health services more actively and has higher levels of health literacy or health anxiety. Accordingly, specialist contact should be interpreted primarily as a marker of overall engagement with the healthcare system and of health-seeking behavior, rather than as an independent mechanism that in itself promotes screening; despite extensive multivariable adjustment, residual confounding by unmeasured behavioral and psychosocial characteristics remains possible. In Andersen’s model of health service utilization behavior, such predisposition and need factors are identified as important determinants of service use [9].

On the other hand, these findings align with existing literature indicating that breast cancer early detection behaviors are multidimensional in nature and are shaped by the interplay of socioeconomic characteristics and access to healthcare services [18,19,20]. Systematic reviews have consistently identified education, income, regular use of healthcare services, and physician recommendation as being among the strongest correlates of screening participation [18,19]. However, it has been reported that identifying socioeconomic disadvantage alone is insufficient, and that contact with the health system is also a critical component in improving screening behaviors [20]. Our study takes this approach a step further; rather than assessing healthcare utilization merely at the level of “utilized/did not utilize,” we separately examined patterns of contact with family physicians/general practitioners and specialist physicians, thereby revealing the relative contributions of these forms of contact to early detection behaviors. Furthermore, through CART analysis—which evaluated sociodemographic characteristics and healthcare contact in conjunction—we identified subgroups of women with significantly differing probabilities of having undergone a recent mammogram. These findings suggest that the quality of contact with the health system, not just individual risk factors, must be considered when fostering early detection behaviors for breast cancer; they also indicate that jointly evaluating sociodemographic characteristics and healthcare utilization data in primary care settings could contribute to the development of targeted counseling strategies and the more effective planning of screening programs.

This study demonstrated that contact with both a family physician/general practitioner and a specialist physician within the past 12 months was independently associated with undergoing recent mammography and performing regular breast self-examination. However, analyses incorporating both contact variables into the same model revealed a stronger association for contact with a specialist physician. A large-scale study conducted in the United States using decision tree analysis reported that having a regular primary care physician and possessing health insurance were the key determinants of mammography uptake [21]. Similarly, Lofters et al. emphasized that the systematic assessment of social determinants in primary care could significantly contribute to identifying women who underutilize screening services [20]. In this study, the fact that health insurance coverage did not emerge as a distinguishing determinant can be considered an expected finding, given that the vast majority of participants (93.7%) possessed social security coverage and mammography services are provided free of charge in the country under the national breast cancer screening program. Conversely, the stronger association observed with specialist physician contact is thought to be linked to factors such as women’s ability to consult specialists directly, the ambiguity regarding whether mammography was performed for diagnostic or screening purposes, the relative ease of access to secondary and tertiary healthcare services, and distinct characteristics of healthcare delivery models. Therefore, we are of the opinion that the relationship between healthcare contact and early breast cancer detection behaviors should not be evaluated independently of the characteristics of the healthcare delivery model. However, due to the cross-sectional design of our study, it is not possible to interpret these relationships in terms of causality.

While multivariate regression models offer a powerful analytical approach for identifying independent determinants of early breast cancer detection behaviors, they may be limited on their own when it comes to defining subgroups of women within the population who share similar risk characteristics [22]. Therefore, in our study, multivariate regression analyses were complemented by CART analysis, and profiles of women with significantly differing probabilities of undergoing recent mammography were identified. In the mammography literature, classification tree analyses have long been used to identify groups of women exhibiting high versus low participation in screening [21,23]. More recently, Pedrós Barnils and Schüz compared regression- and decision tree-based approaches for identifying intersectional groups of women at high risk of non-participation in breast cancer screening; they reported that decision tree analysis could generate more detailed risk profiles than classical regression models and contributed significantly to the identification of target groups [24]. Notably, in this study, the highest normalized importance values in the decision tree model were calculated for any breast cancer screening behavior and contact with a specialist physician. In contrast, contact with a family physician or general practitioner did not appear as an independent splitting variable in the decision tree. This finding suggests that, although contact with a family physician or general practitioner shows an independent association in multivariate models, within the decision tree approach it should be viewed not as a dominant classifier that independently distinguishes women’s early detection behaviors, but rather as a complementary variable that gains significance in conjunction with other individual characteristics and healthcare utilization patterns. Furthermore, Bao et al. demonstrated that, despite organized screening programs increasing participation, socioeconomic disparities have not been entirely eliminated, and screening participation rates remain low among certain subgroups [25]. When these findings are considered together, the use of CART analysis in our study not only went beyond identifying mere independent predictors but also enabled the identification of subgroups of women who exhibit low rates of early detection behavior and could benefit from priority interventions.

The findings of this study indicate that behaviors related to the early detection of breast cancer constitute a multidimensional process shaped not only by individual characteristics but also by the interplay of healthcare service utilization and socioeconomic conditions. Studies evaluating organized breast cancer screening programs in European countries have reported that the mere existence of such programs does not guarantee high participation rates; rather, effective invitation mechanisms, appropriate organizational models, and targeted interventions are decisive factors in screening success [26]. The role assigned to primary care also differs markedly across delivery models. In systems in which organized programmes are built on population registries and centralized invitations—such as those of the United Kingdom, the Netherlands, and the Nordic countries—general practitioners strongly influence attendance through their advisory role even when they do not issue the invitations themselves; in Denmark, for example, women whose general practitioners held more positive attitudes toward mammography screening were more likely to participate [27]. In Türkiye, by contrast, women can access specialist care directly without gatekeeping, invitation mechanisms are less systematically implemented, and an appreciable share of mammography is delivered opportunistically in secondary and tertiary care. This structural difference plausibly explains why specialist contact, rather than primary care contact, dominated the associations observed here, and it cautions against extrapolating our findings directly to gatekeeping-based systems. Taken together, these findings suggest that simply providing screening services is insufficient to foster early detection behaviors; instead, identifying groups of women with varying risk profiles, strengthening engagement with healthcare services, and planning targeted preventive health interventions based on specific needs could significantly enhance the effectiveness of screening programs. In practical terms, several strategies could help translate routine healthcare contact into screening participation in the Turkish context: automated reminder and recall systems linked to family medicine registries; opportunistic delivery of screening recommendations during primary care encounters and, in particular, during chronic disease follow-up visits—a relevant entry point given that women with chronic disease were more likely to have up-to-date mammography; systematic personal invitation letters with pre-scheduled appointments through cancer early diagnosis, screening and training centres (KETEM); and patient navigation for women with low education or income, who consistently showed the lowest screening prevalence. Personal invitation letters, reminders, and the removal of organizational barriers are among the interventions with the strongest evidence of increasing participation in organized screening programmes [28].

Our analytical approach should also be situated within the rapidly evolving field of predictive modelling for early cancer detection. A recent systematic review of machine learning models for supporting early cancer diagnosis in symptomatic patients emphasized that model development, validation, interpretability, and clinical implementation are central to translating predictive tools into practice, and highlighted their potential role in supporting personalized screening strategies and risk stratification [29]. Within this spectrum, CART occupies a deliberately simple and transparent position: its output is a small set of decision rules that can be applied without any computational infrastructure. The tree identified here lends itself to direct use in public health practice: family medicine units and cancer early diagnosis, screening and training centres (KETEM) could use the first two splits—any BSE practice and recent specialist contact—to flag, with minimal effort, women resembling the lowest-uptake profile (women aged 40–49 with neither behavior; mammography prevalence 4.9%) for proactive invitation, counselling, or navigation, while directing reinforcement rather than recruitment efforts toward already-engaged groups. At the same time, the moderate cross-validated discrimination of the model (AUC = 0.681) indicates that a substantial proportion of the variability in screening behavior remains unexplained by the sociodemographic and utilization variables available in the survey; unmeasured determinants—physician recommendation, family history of breast cancer, health literacy, individual risk perception, invitation to organized screening, previous abnormal breast findings, and differential access to preventive services—would likely improve both prediction and understanding, and their absence should temper any use of the present tree as a stand-alone predictive tool.

The retention of monthly BSE as a primary outcome also deserves justification. Randomized evidence has not demonstrated that formal, regular BSE reduces breast cancer mortality, and most international guidelines no longer recommend it as a population screening strategy, refs. [30,31] emphasizing instead breast awareness—familiarity with the normal look and feel of the breast and prompt reporting of changes. In Türkiye, however, monthly BSE remains an explicit component of the National Cancer Control Programme and of public education activities delivered through primary care and KETEM units [7]. In the present analysis, BSE was therefore examined not as a mortality-reducing screening test but as a behavioral indicator of breast awareness and engagement with early detection; consistent with this interpretation, any BSE practice was the strongest single discriminator of mammography uptake in the CART model. The distinction between formal monthly BSE and the broader concept of breast awareness—which our data cannot capture directly—should nevertheless be borne in mind when interpreting this outcome.

## 5. Conclusions

This national population-based study has demonstrated that breast cancer early detection behaviors among women in Türkiye are closely associated with sociodemographic characteristics, health status, and healthcare utilization. In particular, education level and contact with healthcare services—most strongly, contact with a specialist physician—were independently associated with both regular breast self-examination and up-to-date mammography. Because the data are cross-sectional, these associations describe patterns of co-occurrence rather than causal effects, and part of the association with specialist contact may reflect diagnostic pathways and general health-seeking behavior. Our findings reveal that early detection behaviors regarding breast cancer are not uniform across different segments of society and that groups of women with distinct needs must be considered when planning preventive health services. Accordingly, targeted strategies that take into account individual risk profiles and healthcare utilization patterns could contribute to enhancing the effectiveness of national breast cancer screening programs. Future prospective and intervention-based studies would be beneficial in elucidating the causal nature of these relationships and evaluating the effectiveness of targeted screening strategies.

## 6. Strengths and Limitations

This study has certain limitations. First, due to the cross-sectional design of the research, the identified associations cannot be interpreted as causal. In particular, reverse causation—whereby undergoing mammography or experiencing breast symptoms leads to physician contact, rather than the reverse—cannot be excluded. Second, since behaviors regarding mammography and breast self-examination were based on participants’ self-reports, the possibility of recall bias and social desirability bias cannot be ruled out. The timing of the most recent mammography may be especially susceptible to recall error (telescoping), and the resulting misclassification could bias the estimated prevalence of up-to-date mammography in either direction. Furthermore, as the study involved secondary data analysis, certain variables that could influence early detection behaviors—such as family history of breast cancer, individual risk perception, health literacy, physician recommendation, receipt of an invitation for organized screening, whether the mammography was performed for screening or diagnostic purposes, previous abnormal breast findings, and differential access to preventive services—could not be assessed. The inability to separate screening from diagnostic mammography deserves particular emphasis: diagnostic examinations prompted by symptoms are, by definition, preceded by physician contact, and part of the association between specialist contact and mammography is therefore likely to reflect diagnostic pathways rather than screening behavior. Relatedly, because several behavioral and psychosocial determinants of preventive care use were unavailable, residual confounding remains possible, and specialist contact should be viewed in part as a proxy for overall health-seeking behavior. Finally, the moderate discriminatory performance of the CART model (cross-validated AUC = 0.681) indicates that the variables available in the survey explain only part of the variation in screening behavior. Nevertheless, the use of a nationally representative sample, the inclusion of sample weights in the analysis, and the evaluation of early detection behaviors using both multivariate models and complementary analytical approaches are among the study’s significant strengths.

## Figures and Tables

**Table 1:** Characteristics of the analytic sample and early detection behaviors.

Variable	Unweighted n	Weighted %
**Age group**		
40–49	2284	41.3
50–59	1816	32.7
60–69	1453	26.0
**Educational level**		
No formal schooling	990	17.7
Primary school	2688	48.9
Lower secondary school	397	7.1
Upper secondary school (high school)	802	14.3
University or above	676	12.0
**Income group**		
Low	1023	18.1
Middle	2140	39.1
High	2390	42.8
**Self-rated general health**		
Good/very good	2395	43.3
Fair	2488	44.8
Poor/very poor	670	11.9
Married	4410	79.4
Covered by the Social Security Institution (SSI)	5222	93.7
Any chronic disease	4175	75.2
**Healthcare contact in the past 12 months**		
No contact	778	14.7
GP/general practitioner only	925	16.9
Specialist only	670	12.0
Both	3180	56.5
Mammography in the past 12 months	681	12.5
Up-to-date mammography (past 2 years)	1285	23.5
Lifetime mammography	3005	54.4
Monthly BSE	1365	25.0
Any BSE	3192	57.8

Note: Percentages were calculated using the FERTFAKTOR individual sampling weight. BSE, breast self-examination; GP, general practitioner/family physician; SSI, Social Security Institution. Income-group percentages were calculated among participants with non-missing income data. The category “university or above” comprises university and postgraduate levels.

**Table 2:** Frequency of breast self-examination and timing of mammography.

Behavioral Pattern	Unweighted n	Weighted %
**Frequency of BSE**		
Never	2362	42.2
Monthly	1365	25.0
Every 3 months	596	10.9
Less often than every 3 months	1231	21.9
**Timing of the most recent mammography**		
Within the past year	681	12.5
1–2 years ago	604	11.0
2–3 years ago	464	8.5
3–5 years ago	525	9.2
More than 5 years ago	731	13.3
Never	2549	45.6

Note: Original response categories are presented in this table. For the summary indicators, up-to-date mammography was defined as having undergone mammography within the previous 2 years, and monthly BSE as reporting a BSE frequency of once a month.

**Table 3:** Fully adjusted modified Poisson regression models for up-to-date mammography and monthly BSE.

Variable	Reference	Up-to-Date Mammography aPR (95% CI)	*p*	Monthly BSE aPR (95% CI)	*p*
GP only	No contact	0.98 (0.78–1.23)	0.843	1.21 (1.00–1.47)	0.049
Specialist only	No contact	1.38 (1.11–1.72)	0.004	1.41 (1.16–1.72)	<0.001
Both	No contact	1.78 (1.49–2.13)	<0.001	1.52 (1.29–1.79)	<0.001
Age 50–59 years	40–49 years	1.08 (0.97–1.20)	0.178	0.98 (0.88–1.09)	0.718
Age 60–69 years	40–49 years	0.88 (0.77–1.01)	0.067	0.85 (0.75–0.96)	0.009
Primary school	No formal schooling	1.15 (0.98–1.34)	0.083	1.40 (1.19–1.65)	<0.001
Lower secondary school	No formal schooling	1.35 (1.09–1.68)	0.006	1.71 (1.38–2.11)	<0.001
Upper secondary school	No formal schooling	1.37 (1.13–1.65)	<0.001	1.60 (1.32–1.94)	<0.001
University or above	No formal schooling	1.67 (1.38–2.02)	<0.001	1.75 (1.44–2.13)	<0.001
Middle income	Low income	1.07 (0.91–1.25)	0.402	1.23 (1.05–1.45)	0.011
High income	Low income	1.16 (0.99–1.35)	0.072	1.41 (1.20–1.66)	<0.001
Any chronic disease	None	1.22 (1.06–1.40)	0.006	1.08 (0.95–1.23)	0.221

Note: Healthcare contact was entered into the models as a single, mutually exclusive four-category variable based on physician visits within the past 12 months (No contact: women who consulted no physician in the past 12 months). Models were adjusted for age, education, income, marital status, SSI coverage, chronic disease, self-rated general health, tobacco use, alcohol use, e-cigarette experimentation, fruit consumption, vegetable/salad consumption and sleep problems. Weights were derived from FERTFAKTOR and normalised to the sample size. Robust (HC0) variance estimators were used. aPR, adjusted prevalence ratio; CI, confidence interval.

**Table 4:** Fully adjusted associations when GP and specialist contact are entered as two separate indicators.

Contact Variable	Reference	Up-to-Date Mammography aPR (95% CI)	*p*	Monthly BSE aPR (95% CI)	*p*
GP contact (past 12 months)	None	1.18 (1.04–1.33)	0.009	1.12 (1.00–1.26)	0.048
Specialist contact (past 12 months)	None	1.66 (1.46–1.88)	<0.001	1.30 (1.16–1.46)	<0.001

Note: In this model, GP contact and specialist contact were entered into the same model as two separate binary indicators. All other adjustment variables were identical to those in Table 3. This analysis is not equivalent to the “GP only” category of the four-category contact model; it compares all participants with versus without GP contact while adjusting for specialist contact.

## Data Availability

The data employed in this research were of a secondary nature, drawn from the Türkiye Health Survey Research micro datasets provided by the Turkish Statistical Institute. (TÜİK) These datasets are made available to researchers upon request and the payment of a specified fee. In line with the requests of national and international researchers, the Turkish Statistical Institute (TUIK) provides access to and the use of microdata from censuses and surveys within the framework of legal regulations. Detailed information regarding the access procedure for microdata sets and the available microdata can be found on the website https://www.tuik.gov.tr/Kurumsal/Mikro_Veri (accessed on 15 July 2026).

## Abbreviations

The following abbreviations are used in this manuscript:

TUIK	Turkish Statistical Institute
BSE	Breast self-examination
CART	Classification and Regression Tree
